# 6,7-Dimeth­oxy-3-meth­oxy­carbonyl-1-(2-meth­oxy­phen­yl)-3,4-dihydro­isoquinoline 2-oxide

**DOI:** 10.1107/S1600536811016539

**Published:** 2011-05-07

**Authors:** Tricia Naicker, Thavendran Govender, Hendrik G. Kruger, Glenn E. M. Maguire

**Affiliations:** a School of Pharmacy and Pharmacology, University of KwaZulu Natal, Durban 4000, South Africa; b School of Chemistry, University of KwaZulu Natal, Durban 4000, South Africa

## Abstract

In the title compound, C_20_H_21_NO_6_, an *N*-oxide-based organocatalyst, the N-containing six-membered ring adopts a twisted half-chair conformation. No hydrogen bonding or π–π stacking was found within the crystal structure.

## Related literature

For related structures, see: Naicker *et al.* (2010 ▶, 2011 ▶).
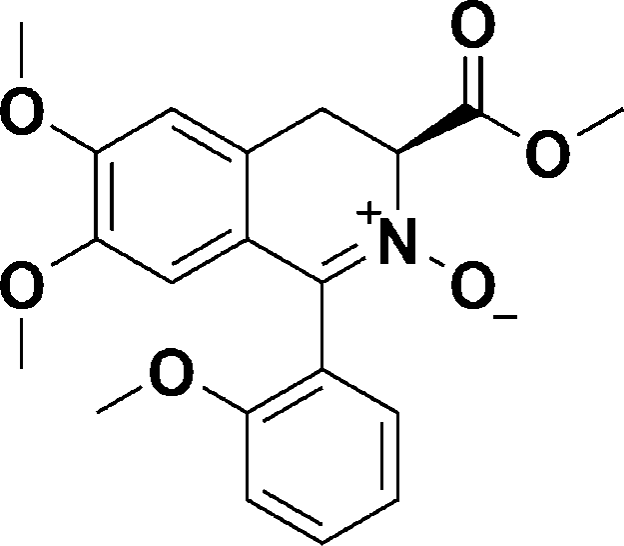


## Experimental

### 

#### Crystal data

C_20_H_21_NO_6_
*M* *_r_* = 371.38
Monoclinic, 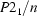

*a* = 5.4765 (1) Å
*b* = 21.9984 (6) Å
*c* = 15.0007 (4) Å
β = 92.774 (2)°
*V* = 1805.08 (8) Å^3^
*Z* = 4
Mo *K*α radiation
μ = 0.10 mm^−1^
*T* = 173 K
0.25 × 0.18 × 0.15 mm

#### Data collection

Bruker APEXII diffractometer
7815 measured reflections
3959 independent reflections
3092 reflections with *I* > 2σ(*I*)
*R*_int_ = 0.016

#### Refinement

*R*[*F* ^2^ > 2σ(*F* ^2^)] = 0.053
*wR*(*F* ^2^) = 0.157
*S* = 1.06
3959 reflections
245 parameters
H-atom parameters constrained
Δρ_max_ = 0.92 e Å^−3^
Δρ_min_ = −0.33 e Å^−3^

### 

Data collection: *APEX2* (Bruker, 2006 ▶); cell refinement: *SAINT* (Bruker, 2006 ▶); data reduction: *SAINT*; program(s) used to solve structure: *SHELXS97* (Sheldrick, 2008 ▶); program(s) used to refine structure: *SHELXL97* (Sheldrick, 2008 ▶); molecular graphics: *OLEX2* (Dolomanov *et al.*, 2009 ▶); software used to prepare material for publication: *SHELXL97*.

## Supplementary Material

Crystal structure: contains datablocks I, global. DOI: 10.1107/S1600536811016539/hg5031sup1.cif

Structure factors: contains datablocks I. DOI: 10.1107/S1600536811016539/hg5031Isup2.hkl

Supplementary material file. DOI: 10.1107/S1600536811016539/hg5031Isup3.cml

Additional supplementary materials:  crystallographic information; 3D view; checkCIF report

## Figures and Tables

**Fig. 1.**
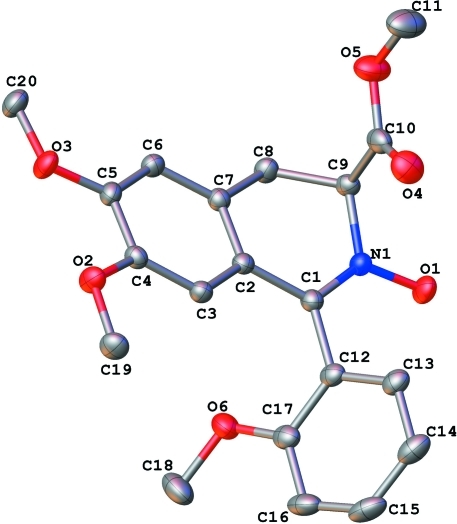
The molecular structure of the title compound with the atom numbering scheme. Displacement ellipsoids are drawn at the 40% probability level. Hydrogen atoms have been omitted for clarity.

**Fig. 2.**
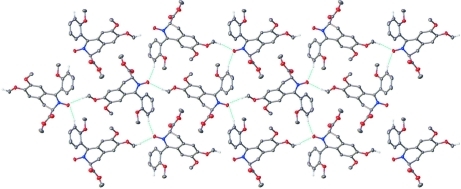
A partial projection of the title compound, viewed along [100] plane.

## supplementary crystallographic information

### Comment

The title compound is a novel *N*-oxide based catalyst containing a tetrahydroisoquinoline (TIQ) backbone. This is the first X-ray crystal structure report of this type of organocatalyst within this TIQ class of molecules. This compound and its derivatives are currently being tested in our laboratory as novel organocatalysts for asymmetric allylation reactions (Naicker *et al.* 2010).

From the crystal structure it is evident that the *N*-containing six membered ring assumes a twisted half chair conformation (Fig. 1). This differs from a similar structure that we recently reported which displays a twisted half boat conformation (Naicker *et al.* 2011). A possible reason for the change is the introduction of the oxygen atom O2 onto the sp2 hybridized nitrogen atom. All our previous examples have either a hydrogen or methyl group at that position.

Interestingly there is no classic hydrogen bonding within the crystal packing however, there are various intermolecular and intramolecular short contact interactions that link the molecules together within the crystal lattice. The *N*-oxide oxygen O2 displays two potential hydrogen bond interactions to C16—H16 and C20A—H20A which are 3.36 Å and 3.34 Å respectively. These interactions result in a layered packing within the crystal stucture as shown along the (100) axis in Fig. 2. A centroid distance of 7.156 Å indicated that there is no π-π stacking within the crystal matrix.

### Experimental

(*S*)-methyl 6,7-dimethoxy-1-(2-methoxyphenyl)-3,4-dihydroisoquinoline-3-carboxylate (1.30 g, 3.7 mmol) was dissolved in dry methylene chloride (50 ml). Potassium carbonate (1.0 g, 7.5 mmol) was added and the reaction cooled to -78 °C. Meta-chloroperbenzoic acid (0.86 g of 75% pure, net 0.65 g, 3.7 mmol) was then added, and the reaction was allowed to stir at -78 °C for 3 h. At this time, the reaction was allowed to warm to room temperature. After stirring for a further 2 h at room temperature, methylene chloride (50 ml) was added to dilute the reaction and celite (500 mg) was added to aid filtration. The reaction was filtered, and the methylene chloride concentrated to dryness affording the crude product which was purified by column chromatography (methylene chloride:methanol, 99:1, *R*_f_ = 1/5) (1.20 g, 87% yield).

Melting point 423 K. [α]^20^_D_ 5.128 (c 0.13 in CHCl_3_).

IR (neat): 2923, 1742, 1508, 1285, 729 cm^-1^.

^1^H NMR (400 MHz, CDCl_3_) δ 7.55 (dd, *J* = 7.5, 1.7 Hz, 1H), 7.51 – 7.42 (m, 1H), 7.20 – 6.97 (m, 3H), 6.74 (d, *J* = 6.5 Hz, 1H), 6.27 (d, *J* = 13.6 Hz, 1H), 4.94 (dt, *J* = 5.8, 2.9 Hz, 1H), 3.90 (d, *J* = 1.8 Hz, 4H), 3.77 (d, *J* = 4.3 Hz, 3H), 3.73 – 3.54 (m, 6H), 3.49 – 3.33 (m, 1H).

^13^C NMR (101 MHz, CDCl_3_) δ 168.74, 157.23, 149.52, 148.17, 131.24, 131.12, 130.43, 122.89, 121.93, 121.49, 121.01, 120.80, 119.71, 112.05, 111.50, 110.64, 110.60, 110.08, 109.50, 77.34, 77.03, 76.71, 70.84, 70.72, 56.17, 56.11, 56.05, 55.93, 55.62, 53.24, 53.10, 30.80, 30.73.

Recrystallization from ethyl acetate at room temperature afforded colourless crystals suitable for X-ray analysis.

### Refinement

All non-hydrogen atoms were refined anisotropically. The hydrogen atoms were placed in idealized positions in a riding model with *U*_iso_ set at 1.2 or 1.5 times those of their parent atoms (1.2 for tertiary C—H, secondary C—H_2_ and aromatic C—H or N—H groups and 1.5 for methyl C—H_3_) and fixed C—H bond lengths(*e.g.* 0.88 Å for N—H and others ranging from 0.95 Å to 1.00 Å).

### Crystal data

**Table:**

C_20_H_21_NO_6_	*F*(000) = 784
*M**_r_* = 371.38	*D*_x_ = 1.367 Mg m^−^^3^
Monoclinic, *P*2_1_/*n*	Melting point: 423 K
Hall symbol: -P 2yn	Mo *K*α radiation, λ = 0.71073 Å
*a* = 5.4765 (1) Å	Cell parameters from 7815 reflections
*b* = 21.9984 (6) Å	θ = 2.3–27.1°
*c* = 15.0007 (4) Å	µ = 0.10 mm^−^^1^
β = 92.774 (2)°	*T* = 173 K
*V* = 1805.08 (8) Å^3^	Block, colourless
*Z* = 4	0.25 × 0.18 × 0.15 mm

### Data collection

**Table:**

Bruker APEXII diffractometer	3092 reflections with *I* > 2σ(*I*)
Radiation source: fine-focus sealed tube	*R*_int_ = 0.016
graphite	θ_max_ = 27.1°, θ_min_ = 2.3°
1.2° φ and ω scans	*h* = −7→7
7815 measured reflections	*k* = −28→28
3959 independent reflections	*l* = −19→19

### Refinement

**Table:**

Refinement on *F*^2^	Secondary atom site location: difference Fourier map
Least-squares matrix: full	Hydrogen site location: inferred from neighbouring sites
*R*[*F*^2^ > 2σ(*F*^2^)] = 0.053	H-atom parameters constrained
*wR*(*F*^2^) = 0.157	*w* = 1/[σ^2^(*F*_o_^2^) + (0.080*P*)^2^ + 1.0977*P*] where *P* = (*F*_o_^2^ + 2*F*_c_^2^)/3
*S* = 1.06	(Δ/σ)_max_ < 0.001
3959 reflections	Δρ_max_ = 0.92 e Å^−^^3^
245 parameters	Δρ_min_ = −0.33 e Å^−^^3^
0 restraints	Extinction correction: *SHELXL97* (Sheldrick, 2008), Fc^*^=kFc[1+0.001xFc^2^λ^3^/sin(2θ)]^-1/4^
Primary atom site location: structure-invariant direct methods	Extinction coefficient: 0.0055 (19)

### Special details

**Table:**

Experimental. Half sphere of data collected using *COLLECT* strategy (Nonius, 2000). Crystal to detector distance = 30 mm; combination of φ and ω scans of 1.0°, 40 s per °, 2 iterations.
Geometry. All e.s.d.'s (except the e.s.d. in the dihedral angle between two l.s. planes) are estimated using the full covariance matrix. The cell e.s.d.'s are taken into account individually in the estimation of e.s.d.'s in distances, angles and torsion angles; correlations between e.s.d.'s in cell parameters are only used when they are defined by crystal symmetry. An approximate (isotropic) treatment of cell e.s.d.'s is used for estimating e.s.d.'s involving l.s. planes.
Refinement. Refinement of *F*^2^ against ALL reflections. The weighted *R*-factor *wR* and goodness of fit *S* are based on *F*^2^, conventional *R*-factors *R* are based on *F*, with *F* set to zero for negative *F*^2^. The threshold expression of *F*^2^ > σ(*F*^2^) is used only for calculating *R*-factors(gt) *etc*. and is not relevant to the choice of reflections for refinement. *R*-factors based on *F*^2^ are statistically about twice as large as those based on *F*, and *R*- factors based on ALL data will be even larger.

### Fractional atomic coordinates and isotropic or equivalent isotropic displacement parameters (Å^2^)

**Table:**

	*x*	*y*	*z*	*U*_iso_*/*U*_eq_	
O1	0.2176 (3)	0.22907 (6)	0.35593 (9)	0.0354 (3)	
O2	0.6006 (2)	−0.03624 (6)	0.11617 (9)	0.0322 (3)	
O3	0.2824 (3)	−0.10179 (6)	0.19301 (9)	0.0344 (3)	
O4	0.5317 (3)	0.12825 (8)	0.45676 (10)	0.0441 (4)	
O5	0.2474 (3)	0.07631 (8)	0.52628 (10)	0.0485 (4)	
O6	0.2093 (3)	0.16305 (7)	0.07924 (9)	0.0391 (4)	
N1	0.2146 (3)	0.17212 (7)	0.33291 (10)	0.0251 (3)	
C1	0.3125 (3)	0.14938 (8)	0.26149 (11)	0.0246 (4)	
C2	0.3023 (3)	0.08366 (8)	0.24573 (11)	0.0228 (4)	
C3	0.4608 (3)	0.05630 (8)	0.18643 (11)	0.0247 (4)	
H3	0.5749	0.0806	0.1567	0.030*	
C4	0.4517 (3)	−0.00558 (8)	0.17115 (11)	0.0246 (4)	
C5	0.2793 (3)	−0.04178 (8)	0.21365 (12)	0.0260 (4)	
C6	0.1259 (3)	−0.01473 (8)	0.27287 (12)	0.0262 (4)	
H6	0.0115	−0.0390	0.3025	0.031*	
C7	0.1370 (3)	0.04758 (8)	0.28959 (11)	0.0238 (4)	
C8	−0.0255 (3)	0.07830 (8)	0.35355 (12)	0.0283 (4)	
H8A	−0.0748	0.0488	0.3991	0.034*	
H8B	−0.1752	0.0932	0.3210	0.034*	
C9	0.1083 (3)	0.13129 (8)	0.39883 (12)	0.0259 (4)	
H9	−0.0107	0.1550	0.4335	0.031*	
C10	0.3260 (4)	0.11235 (9)	0.46255 (13)	0.0344 (5)	
C11	0.4434 (5)	0.06056 (13)	0.59251 (17)	0.0572 (7)	
H11A	0.3780	0.0341	0.6382	0.086*	
H11B	0.5086	0.0978	0.6206	0.086*	
H11C	0.5744	0.0393	0.5629	0.086*	
C12	0.4564 (3)	0.19020 (8)	0.20530 (12)	0.0278 (4)	
C13	0.6553 (3)	0.22127 (8)	0.24385 (14)	0.0314 (4)	
H13	0.6881	0.2188	0.3065	0.038*	
C14	0.8070 (4)	0.25591 (9)	0.19251 (17)	0.0410 (5)	
H14	0.9427	0.2770	0.2195	0.049*	
C15	0.7571 (4)	0.25908 (10)	0.10160 (17)	0.0455 (6)	
H15	0.8616	0.2823	0.0660	0.055*	
C16	0.5593 (4)	0.22943 (10)	0.06094 (15)	0.0418 (5)	
H16	0.5270	0.2328	−0.0017	0.050*	
C17	0.4075 (4)	0.19452 (8)	0.11258 (13)	0.0323 (4)	
C18	0.1512 (5)	0.16733 (12)	−0.01499 (14)	0.0520 (6)	
H18A	0.0059	0.1428	−0.0303	0.078*	
H18B	0.2892	0.1523	−0.0479	0.078*	
H18C	0.1187	0.2098	−0.0311	0.078*	
C19	0.7699 (4)	−0.00092 (10)	0.06834 (13)	0.0351 (5)	
H19A	0.8650	−0.0278	0.0311	0.053*	
H19B	0.8806	0.0207	0.1108	0.053*	
H19C	0.6803	0.0286	0.0303	0.053*	
C20	0.0983 (4)	−0.13944 (9)	0.22980 (15)	0.0364 (5)	
H20A	0.1191	−0.1815	0.2100	0.055*	
H20B	−0.0640	−0.1248	0.2094	0.055*	
H20C	0.1140	−0.1378	0.2951	0.055*	

### Atomic displacement parameters (Å^2^)

**Table:**

	*U*^11^	*U*^22^	*U*^33^	*U*^12^	*U*^13^	*U*^23^
O1	0.0496 (8)	0.0207 (7)	0.0370 (7)	−0.0003 (6)	0.0121 (6)	−0.0065 (6)
O2	0.0374 (7)	0.0271 (7)	0.0335 (7)	0.0029 (6)	0.0143 (6)	−0.0035 (5)
O3	0.0425 (8)	0.0194 (6)	0.0422 (8)	−0.0021 (6)	0.0122 (6)	−0.0042 (6)
O4	0.0335 (8)	0.0543 (10)	0.0441 (9)	0.0016 (7)	−0.0020 (6)	−0.0001 (7)
O5	0.0505 (10)	0.0559 (10)	0.0386 (8)	−0.0023 (8)	−0.0030 (7)	0.0157 (7)
O6	0.0542 (9)	0.0366 (8)	0.0261 (7)	0.0019 (7)	−0.0020 (6)	0.0010 (6)
N1	0.0295 (8)	0.0213 (7)	0.0247 (7)	−0.0006 (6)	0.0040 (6)	−0.0014 (6)
C1	0.0292 (9)	0.0223 (8)	0.0223 (8)	0.0019 (7)	0.0024 (7)	−0.0001 (7)
C2	0.0256 (9)	0.0225 (8)	0.0202 (8)	0.0007 (7)	0.0006 (6)	0.0006 (6)
C3	0.0279 (9)	0.0240 (9)	0.0225 (8)	−0.0005 (7)	0.0038 (7)	0.0002 (7)
C4	0.0275 (9)	0.0256 (9)	0.0211 (8)	0.0031 (7)	0.0034 (7)	−0.0019 (7)
C5	0.0315 (9)	0.0203 (8)	0.0261 (9)	0.0002 (7)	−0.0003 (7)	−0.0012 (7)
C6	0.0277 (9)	0.0250 (9)	0.0260 (9)	−0.0032 (7)	0.0033 (7)	0.0002 (7)
C7	0.0251 (8)	0.0240 (9)	0.0225 (8)	0.0010 (7)	0.0016 (6)	−0.0010 (7)
C8	0.0286 (9)	0.0269 (9)	0.0299 (9)	−0.0014 (7)	0.0071 (7)	−0.0023 (7)
C9	0.0304 (9)	0.0247 (9)	0.0230 (9)	0.0025 (7)	0.0074 (7)	0.0003 (7)
C10	0.0522 (13)	0.0269 (10)	0.0245 (9)	0.0061 (9)	0.0068 (8)	−0.0033 (7)
C11	0.0544 (15)	0.0673 (17)	0.0485 (14)	−0.0081 (13)	−0.0119 (11)	0.0216 (13)
C12	0.0350 (10)	0.0195 (8)	0.0294 (9)	0.0038 (7)	0.0081 (7)	0.0010 (7)
C13	0.0341 (10)	0.0227 (9)	0.0379 (10)	0.0024 (7)	0.0063 (8)	0.0016 (8)
C14	0.0370 (11)	0.0267 (10)	0.0603 (14)	−0.0005 (8)	0.0124 (10)	0.0053 (9)
C15	0.0492 (13)	0.0313 (11)	0.0583 (14)	0.0040 (9)	0.0258 (11)	0.0149 (10)
C16	0.0611 (14)	0.0319 (11)	0.0339 (11)	0.0092 (10)	0.0179 (10)	0.0106 (9)
C17	0.0436 (11)	0.0242 (9)	0.0296 (10)	0.0070 (8)	0.0071 (8)	0.0017 (7)
C18	0.0755 (17)	0.0523 (14)	0.0275 (11)	0.0137 (13)	−0.0059 (11)	−0.0040 (10)
C19	0.0360 (11)	0.0378 (11)	0.0325 (10)	0.0011 (8)	0.0132 (8)	−0.0016 (8)
C20	0.0423 (11)	0.0236 (9)	0.0438 (12)	−0.0061 (8)	0.0059 (9)	−0.0007 (8)

### Geometric parameters (Å, °)

**Table:**

O1—N1	1.2995 (19)	C8—H8B	0.9900
O2—C4	1.366 (2)	C9—C10	1.549 (3)
O2—C19	1.429 (2)	C9—H9	1.0000
O3—C5	1.356 (2)	C11—H11A	0.9800
O3—C20	1.436 (2)	C11—H11B	0.9800
O4—C10	1.187 (3)	C11—H11C	0.9800
O5—C10	1.329 (3)	C12—C13	1.388 (3)
O5—C11	1.468 (3)	C12—C17	1.407 (3)
O6—C17	1.362 (3)	C13—C14	1.388 (3)
O6—C18	1.437 (2)	C13—H13	0.9500
N1—C1	1.320 (2)	C14—C15	1.380 (3)
N1—C9	1.476 (2)	C14—H14	0.9500
C1—C2	1.466 (2)	C15—C16	1.381 (4)
C1—C12	1.484 (2)	C15—H15	0.9500
C2—C7	1.393 (2)	C16—C17	1.394 (3)
C2—C3	1.408 (2)	C16—H16	0.9500
C3—C4	1.381 (2)	C18—H18A	0.9800
C3—H3	0.9500	C18—H18B	0.9800
C4—C5	1.411 (3)	C18—H18C	0.9800
C5—C6	1.386 (3)	C19—H19A	0.9800
C6—C7	1.394 (2)	C19—H19B	0.9800
C6—H6	0.9500	C19—H19C	0.9800
C7—C8	1.501 (2)	C20—H20A	0.9800
C8—C9	1.520 (2)	C20—H20B	0.9800
C8—H8A	0.9900	C20—H20C	0.9800
			
C4—O2—C19	117.10 (15)	O5—C11—H11A	109.5
C5—O3—C20	117.19 (15)	O5—C11—H11B	109.5
C10—O5—C11	112.08 (18)	H11A—C11—H11B	109.5
C17—O6—C18	117.57 (18)	O5—C11—H11C	109.5
O1—N1—C1	125.52 (15)	H11A—C11—H11C	109.5
O1—N1—C9	114.13 (14)	H11B—C11—H11C	109.5
C1—N1—C9	120.15 (15)	C13—C12—C17	119.13 (18)
N1—C1—C2	119.40 (16)	C13—C12—C1	119.27 (16)
N1—C1—C12	118.66 (15)	C17—C12—C1	121.42 (17)
C2—C1—C12	121.51 (15)	C12—C13—C14	121.2 (2)
C7—C2—C3	119.32 (16)	C12—C13—H13	119.4
C7—C2—C1	120.45 (16)	C14—C13—H13	119.4
C3—C2—C1	120.23 (16)	C15—C14—C13	118.7 (2)
C4—C3—C2	120.54 (16)	C15—C14—H14	120.6
C4—C3—H3	119.7	C13—C14—H14	120.6
C2—C3—H3	119.7	C14—C15—C16	121.8 (2)
O2—C4—C3	124.72 (16)	C14—C15—H15	119.1
O2—C4—C5	115.23 (15)	C16—C15—H15	119.1
C3—C4—C5	120.05 (16)	C15—C16—C17	119.4 (2)
O3—C5—C6	125.37 (16)	C15—C16—H16	120.3
O3—C5—C4	115.48 (16)	C17—C16—H16	120.3
C6—C5—C4	119.13 (16)	O6—C17—C16	124.20 (18)
C5—C6—C7	121.00 (16)	O6—C17—C12	116.05 (17)
C5—C6—H6	119.5	C16—C17—C12	119.8 (2)
C7—C6—H6	119.5	O6—C18—H18A	109.5
C2—C7—C6	119.92 (16)	O6—C18—H18B	109.5
C2—C7—C8	117.68 (15)	H18A—C18—H18B	109.5
C6—C7—C8	122.40 (16)	O6—C18—H18C	109.5
C7—C8—C9	110.07 (15)	H18A—C18—H18C	109.5
C7—C8—H8A	109.6	H18B—C18—H18C	109.5
C9—C8—H8A	109.6	O2—C19—H19A	109.5
C7—C8—H8B	109.6	O2—C19—H19B	109.5
C9—C8—H8B	109.6	H19A—C19—H19B	109.5
H8A—C8—H8B	108.2	O2—C19—H19C	109.5
N1—C9—C8	111.39 (14)	H19A—C19—H19C	109.5
N1—C9—C10	105.07 (14)	H19B—C19—H19C	109.5
C8—C9—C10	114.19 (15)	O3—C20—H20A	109.5
N1—C9—H9	108.7	O3—C20—H20B	109.5
C8—C9—H9	108.7	H20A—C20—H20B	109.5
C10—C9—H9	108.7	O3—C20—H20C	109.5
O4—C10—O5	124.7 (2)	H20A—C20—H20C	109.5
O4—C10—C9	125.50 (18)	H20B—C20—H20C	109.5
O5—C10—C9	109.75 (18)		
			
O1—N1—C1—C2	177.89 (16)	O1—N1—C9—C8	146.52 (15)
C9—N1—C1—C2	3.4 (2)	C1—N1—C9—C8	−38.4 (2)
O1—N1—C1—C12	5.3 (3)	O1—N1—C9—C10	−89.34 (17)
C9—N1—C1—C12	−169.18 (15)	C1—N1—C9—C10	85.72 (18)
N1—C1—C2—C7	17.8 (2)	C7—C8—C9—N1	51.46 (19)
C12—C1—C2—C7	−169.84 (16)	C7—C8—C9—C10	−67.4 (2)
N1—C1—C2—C3	−161.82 (16)	C11—O5—C10—O4	3.0 (3)
C12—C1—C2—C3	10.6 (3)	C11—O5—C10—C9	−175.79 (18)
C7—C2—C3—C4	0.5 (3)	N1—C9—C10—O4	−0.3 (3)
C1—C2—C3—C4	−179.88 (16)	C8—C9—C10—O4	122.1 (2)
C19—O2—C4—C3	−2.9 (3)	N1—C9—C10—O5	178.51 (15)
C19—O2—C4—C5	176.99 (16)	C8—C9—C10—O5	−59.1 (2)
C2—C3—C4—O2	−178.80 (16)	N1—C1—C12—C13	56.9 (2)
C2—C3—C4—C5	1.3 (3)	C2—C1—C12—C13	−115.55 (19)
C20—O3—C5—C6	5.5 (3)	N1—C1—C12—C17	−128.06 (19)
C20—O3—C5—C4	−175.62 (16)	C2—C1—C12—C17	59.5 (2)
O2—C4—C5—O3	−1.0 (2)	C17—C12—C13—C14	−0.6 (3)
C3—C4—C5—O3	178.87 (16)	C1—C12—C13—C14	174.57 (17)
O2—C4—C5—C6	177.95 (15)	C12—C13—C14—C15	0.0 (3)
C3—C4—C5—C6	−2.2 (3)	C13—C14—C15—C16	0.8 (3)
O3—C5—C6—C7	−179.98 (17)	C14—C15—C16—C17	−1.0 (3)
C4—C5—C6—C7	1.2 (3)	C18—O6—C17—C16	−1.3 (3)
C3—C2—C7—C6	−1.5 (2)	C18—O6—C17—C12	178.88 (18)
C1—C2—C7—C6	178.87 (16)	C15—C16—C17—O6	−179.39 (19)
C3—C2—C7—C8	178.87 (15)	C15—C16—C17—C12	0.4 (3)
C1—C2—C7—C8	−0.7 (2)	C13—C12—C17—O6	−179.81 (16)
C5—C6—C7—C2	0.7 (3)	C1—C12—C17—O6	5.1 (3)
C5—C6—C7—C8	−179.74 (16)	C13—C12—C17—C16	0.4 (3)
C2—C7—C8—C9	−33.2 (2)	C1—C12—C17—C16	−174.66 (17)
C6—C7—C8—C9	147.19 (17)		
